# Substance-in-Use Data Sheets for Undergraduate Synthesis Experiments

**DOI:** 10.1021/acs.jchemed.4c00974

**Published:** 2025-02-03

**Authors:** Vladimir L. Kolesnichenko, Galina Z. Goloverda

**Affiliations:** Xavier University of Louisiana, Chemistry Department, 1 Drexel Dr., New Orleans, Louisiana 70125, United States

**Keywords:** Lab Safety, Safe Practice, Chemical Handling, Descriptive Chemistry, Cognitive Learning

## Abstract

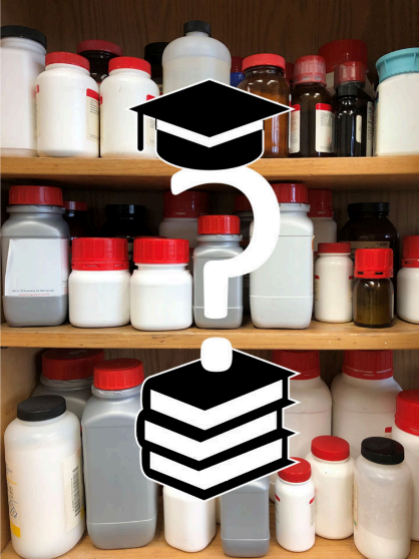

Students in upper-level undergraduate lab classes are required to collect data on the basic properties and hazard information on chemicals used in their upcoming experiments. Based on this information, which is summarized in a provided one-page Substance-in-use Data Sheet, students compose a “Handling Techniques” section of the sheet. This is submitted 1 day before the experiment for instructor evaluation and approval and for in-class discussion. Finding and systematically organizing this information helps students handle chemicals and conduct reactions more consciously and safely. It also enhances their cognitive learning, contributes to their foundational chemistry knowledge through practical experience, and helps develop the experimental science skills essential to future professionals.

One of the most important things in teaching an undergraduate chemistry lab is unquestionably safety, which is closely related to the students’ in-depth familiarity with the chemicals they are about to handle, their properties, and hazards these chemicals may pose. It is believed that shaping the students’ attitudes to lab safety should start during undergraduate education and safety training should be incorporated in the undergraduate curriculum.^1−9^ Recent reviews and critiques in *Nature Chemistry*(10) call for re-examination of safety approaches due to a substantial increase in major accidents in chemistry laboratories worldwide in recent years. This issue has become even more important in “post-COVID-19” higher level lab classes, as some students missed a hands-on experience by taking their freshmen and sophomore laboratories online and learning lab and safety techniques from videos during the pandemic. While some of these videos are well-done, they may create an impression that chemistry laboratory techniques can be learned without having hands-on experience, leading to even more safety hazards as these students progress in their academic or professional careers. Pedagogic goals, learning criteria, and the value of laboratory experience in chemistry education have been extensively discussed within the academic community.^11,12^

Traditionally, the hazard information about a chemical can be found in Material Safety Data Sheets (MSDSs), which are currently called Safety Data Sheets (SDSs). These sources contain a lot of essential information but are often not well organized for undergraduates; they are too long and contain much irrelevant information and sometimes even erroneous statements.^13^ The Department of Health web pages of some US states offer valuable guidelines on handling chemicals.^14^ Nelson suggests an introduction of a student exercise involving preparation of a one-page experiment-specific Laboratory Chemical Safety Summary (LCSS),^15^ based on summaries presented in *Prudent Practices in the Laboratory*(16) and MSDS sheets. Despite various regulations and guidelines, a recent assessment of the laboratory safety training in undergraduate education,^17^ via a survey completed by first-year doctoral students, revealed deficiencies in undergraduate safety education. The assessment identified that a methodological approach, specific training methods, and “frequent high-quality safety analyses of prospective experiments are essential”.

Typically, in a chemistry lab class, students are given detailed instruction for the experiment, including techniques, chemical handling, and safety notes. These instructions are usually presented as a list of do’s and don’ts without logical explanation. A careful student, who follows all of the guidelines, will likely succeed with the assigned routine, but such a student may have little motivation to turn this experience into a skill because the practical aspects of chemistry are often not assessed. Having only cookbook-like instruction may also be a missed opportunity for students to learn about the properties of unfamiliar reagents or solvents in a broader scope than that of the assigned reaction and thus to enhance the connection between theoretical concepts and practical experience.

Consider, for example, the bromination of toluene. The exact protocol is given to students who may or may not read it before class. They already know the utility and mechanism of this electrophilic substitution reaction, but they have never seen elemental bromine, toluene, or aluminum bromide (or chloride) and do not know about their chemical and physical properties. Are they all solids, liquids, or gases? The instructions most likely suggest handling bromine in the fume hood but do not mention much about protecting laboratory equipment from its corrosive fumes. What should they do if the routine goes awry? How should they neutralize a spill? What should they expect if bromine penetrates a damaged glove? Why is toluene used instead of classical benzene for this experiment? Do the gloves protect well if these liquids are on their hands? What happens to the aluminum bromide (or chloride) catalyst if it is exposed to moist air? What happens to the leftovers of each reagent and the reaction byproducts during the workup procedure? Can this same technique be adopted for bromination of other substrates such as acetophenone? (Box 1).

Box 1 Using a catalytic amount of AlBr_3_ or AlCl_3_ promotes bromination of the methyl group, yielding phenacyl bromide, while 3-bromoacetophenone can form only if the amount of catalyst exceeds stoichiometric amounts.

In this paper, we describe the author’s experience in teaching an upper-level chemistry lab course, demonstrating that students substantially benefitted from homework assignments preceding each synthesis experiment. Each student was required to collect information about the basic properties of the chemicals used in the forthcoming experiment and fill out a one-page Substance-in-use Data Sheet. Based on the properties, students were supposed to decide and answer questions on how to handle each chemical, including the reaction workup, spill removals and disposal, and glassware cleaning. Itemized blank Data Sheets of three types are provided to students: one for molecular compounds (Table 1), one for ionic compounds, and one for elements (see the Supporting Information). We did not make a separate data sheet for covalent network compounds because they are not as numerous in everyday synthesis practice and can be described similarly to ionic compounds.

**Table 1:**
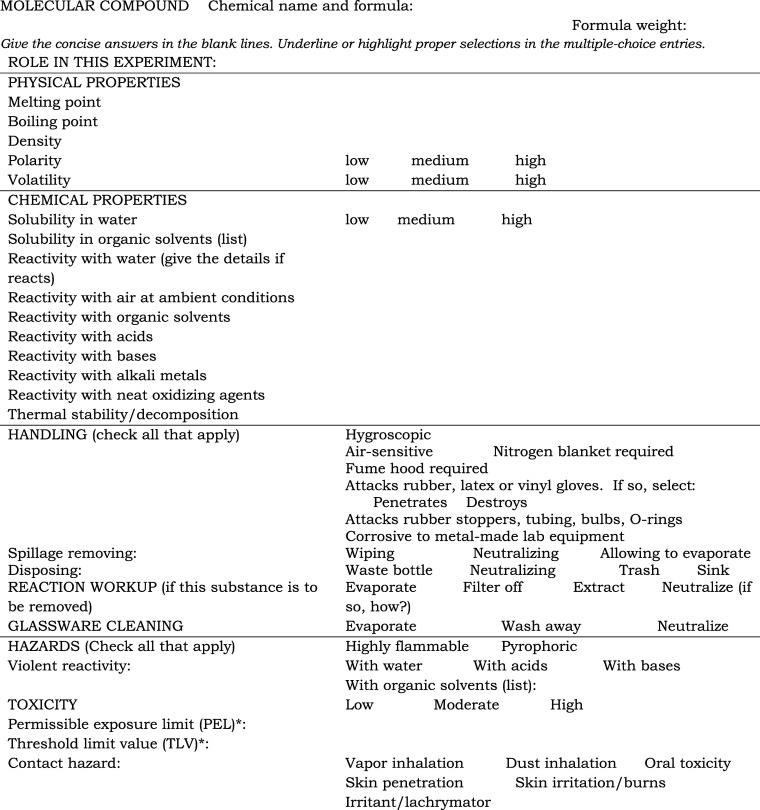
Substance-in-Use Blank Form for Molecular Compounds

* Refer to https://www.osha.gov/annotated-pels or other sources.

In modern chemistry education, the descriptive component, once prevalent, has tended to gradually fade and is sometimes diminished or absent. The removal of this aspect risks weakening the connection between chemistry courses and the experimental nature of the discipline. Finding and systematically organizing information for the properties section of the Substance-in-use Data Sheets enhances students’ cognitive learning and strengthens their fundamental chemistry knowledge through practical experience. This approach also promotes more mindful and safe chemical handling, enabling students to perform their synthesis routines with greater awareness and care.

The first section of the Substance-in-use Data Sheet asks for the role of the substance in the synthesis, followed by its physical and chemical properties. Physical properties to be listed include melting and boiling points, density and volatility for molecular compounds and elements, and polarity for molecular compounds. Chemical properties include the reactivity of a chemical with water, oxygen, halogens, acids, bases, metals, oxidizing agents, and organic solvents, as well as its solubility in water and organic solvents. Only general descriptive information is summarized in this section (Box 2).

Box 2 The criteria for polarity: permittivity <10 – low, 10–20 – medium, >20 – high; for volatility: normal boiling point <100 – high, 100–200 – medium, >200 – low; for solubility g/L: <1 – low, 1–20 – medium, >20 – high.

The next section of the Substance-in-use Data Sheets is entirely practical, requesting guidance on handling, disposal methods, reaction workup, glassware cleaning, and hazard information. Handling techniques are logically derived from physical and chemical properties as well as from environmental hazard and toxicity data. It is expected that most items in this section are composed by the students themselves (Box 3).

Box 3 The data sheet for AlBr_3_ used as a catalyst for electrophilic bromination of aromatic substrates would have the following entry for the reaction workup: aluminum bromide (or chloride) catalyst has to be neutralized first by reacting it with water, which causes its dissociation and hydrolysis. The data sheet for Br_2_ would contain the following entry: bromine spill is best neutralized with aqueous sodium thiosulfate, which reduces it to bromide.

Students collect information outside of the class time, and they are free to use any reliable information sources including textbooks, handbooks, lab manuals, encyclopedias, and review articles. Initially, submitting a list of references was not required, but students were encouraged to keep it handy and to be ready to refer to their sources if requested.

The Substance-in-use Data Sheets for each defined by instructor compound were required to be turned in at least 1 day before the experiment, and their submission was set as a passcode to the lab. The main emphasis of this home work was not on collecting the information itself but on the ability of a student to analyze it and make conclusions with regard to practical handling of a chemical and its proper disposal. Based on information in a descriptive section of the Substance-in-use Data Sheets consisting of encyclopedic data on physical, chemical, and toxicity properties, which are seldom inaccurate, students were supposed to compose a practical section of the sheets. This section included handling, removal (separation or neutralization), disposal, reaction workup, and glassware cleaning, all related to the chemical’s properties, and it was graded. Regarding waste disposal, students were expected to identify one of the general categories listed in the Data Sheet, namely, a designated waste bottle, neutralization, drain, or trash for nonhazardous waste. The Data Sheets’ grade comprised 8% of the total grade in the class.

Prior to the beginning of the experiment, all answers for each chemical were discussed and corrected as necessary. Incorrect answers were identified by the instructor ahead of the discussion, and students were expected to reveal the source of inaccurate information. Specific guidelines on handling chemicals, reaction workup, spill cleanup, and waste disposal methods were provided to students in the prelab instruction. Chemical waste was categorized into groups such as halogenated or nonhalogenated solid or liquid organics and chemically inert, nonvolatile substances that are toxic for the environment, with each category collected in separate bottles. Highly reactive substances were neutralized using specific methods prior to disposal. The importance of chemical compatibility was emphasized as a critical topic in the context of waste disposal.

As an example, a typical data sheet for toluene composed by a student and reviewed by an instructor is shown in Table 2 (student’s choices are underlined, and the verbal answers are in *italic*). During a prelab in-class discussion, the instructor goes over the Substance-in-use Data Sheets for each of the assigned compounds and briefly comments on the criteria for making correct choices. For instance, relatively low melting and boiling points are generally attributed to low molecular weight molecular compounds, so the volatility of toluene should be marked as medium. Since toluene is a hydrocarbon, its density and polarity should be marked as low. Low polarity is attributed to low solubility in water (having no heteroatoms, O or N) but high miscibility with organic solvents. As a consequence of the high thermodynamic stability of C–H and C–C bonds, toluene has a low reactivity with common reagents such as water, dilute acids, bases, and alkali metals. Like all hydrocarbons, toluene is easily oxidizable with oxygen or condensed-phase oxidizing agents (its Δ_c_*H°* = 3910 kJ/mol); however, a relatively high activation barrier for its combustion makes this reaction extremely slow at ambient conditions. Therefore, the corresponding lines in the Substance-in-use Data Sheet should be marked none, but the line in the hazard section should be marked highly flammable.

**Table 2:**
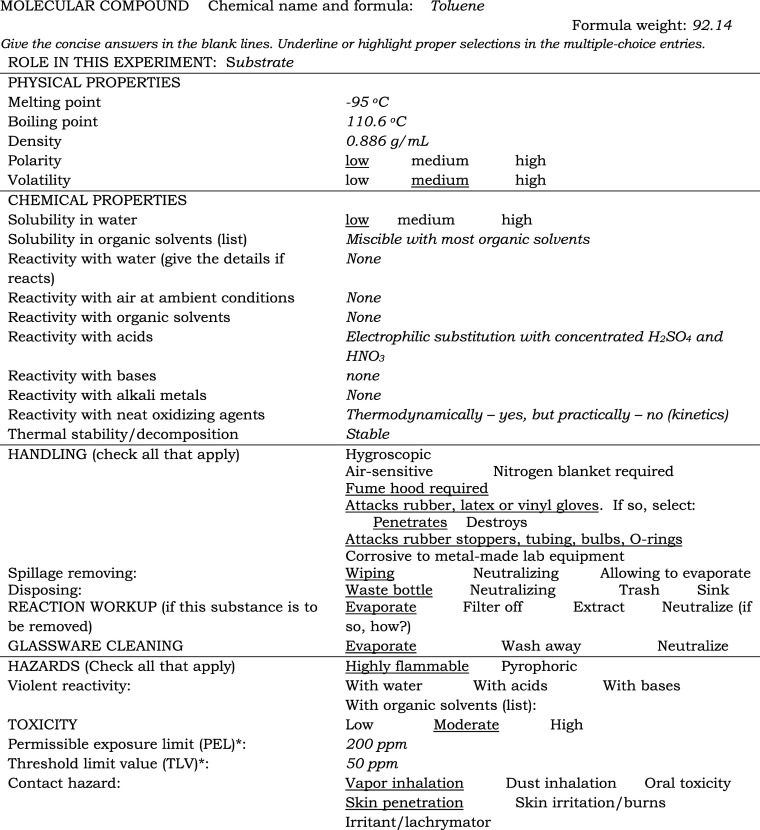
Substance-in-Use Data Sheet for Toluene (Composed by a Student)

Selections in the HANDLING section are determined by all of the above plus the toxicity data. Since this section is composed by students, it is carefully reviewed and redelivered to them. The rationale might look as follows: relatively high toxicity, volatility, and flammability suggest using a fume hood and a waste bottle for nonhalogenated flammables for disposal. The relatively high volatility of toluene indicates selecting evaporation in the reaction workup and glassware cleaning sections. Vapor inhalation and skin penetration are likely paths associated with human exposure. The low polarity and volatility of toluene contribute to its damaging effect on rubber (stoppers, tubing, and O-rings) and its diffusion through latex gloves.

It is also communicated to students that using toluene instead of benzene as a solvent or reagent is preferred whenever possible because benzene is 20 times more toxic than toluene. While latex gloves are not effective barriers against these nonpolar fluids, the current version of the SDS for toluene does not specify the type of gloves to be used. Proper glove selection can be (optionally) determined from multiple online suppliers’ web pages. More examples of the completed data sheets are provided in the Supporting Information.

Questions on the Substance-in-use Data Sheets were incorporated into the postexperiment quizzes (see the Supporting Information). The focus was on the students’ ability to connect the properties of compounds to their applications in the lab. Emphasis was also placed on practical handling techniques in relation to the compounds’ physical, chemical, and toxicity properties. Students were expected to demonstrate their ability to generalize compound types, properties, and handling practices and apply these relationships to particular classes of compounds. Questions on Data Sheets can be also included in the final exam in a more general form (see the Supporting Information).

Statistics on students’ performance collected in the course of several years shows that creating the Substance-in-use Data Sheets and using them for in-depth discussions boosted their overall class performance. Most students exhibited improved familiarity with the chemicals they handled and increased awareness of the associated hazards, which was evident from their benchtop work confidence, quality and quickness, and a good postexperiment quiz experience. The authors observed a 14 percent final grade increase (however, some other factors could also contribute to this change).

Having the Substance-in-use Data Sheets as a solid requirement helped students work confidently, safely, and efficiently. This experience addresses many questions raised in recent discussions.^11,12^ Searching the chemistry literature, extracting relevant information, assembling it in a proper format, and using it to compose handling instructions are potent prelab activities that enhance the learning of chemistry and help develop the experimental science skills essential for future professionals.

Learning ahead of time about unfamiliar chemicals is expected to become a valuable habit that can be carried far beyond undergraduate classes. In fact, it is a solid habit of the authors of this article in their research experience. We recommend that Substance-in-use Data Sheets be introduced for all undergraduate chemistry lab classes dealing with synthesis and adjusted appropriately for the class level.

## Supporting Information Available

Substance-in-use blank forms for ionic compounds and elements, examples of Substance-in-use data sheets completed by students, selected quiz questions, and selected test questions (PDF)
