# Supplementary material for: Substance-in-Use Data Sheets for Undergraduate Synthesis Experiments
Source: J Chem Educ. 2025 Feb 3;102(3):1071–5. doi: 10.1021/acs.jchemed.4c00974 (PMC11905282; doi:10.1021/acs.jchemed.4c00974)
Supplement: Supplementary file 1 — ed4c00974_si_001.pdf [file ed4c00974_si_001.pdf]

SUBSTANCE-IN-USE DATA SHEETS FOR UNDERGRADUATE SYNTHESIS EXPERIMENTS

Vladimir L. Kolesnichenko* and Galina Z. Goloverda*

E-mail vkolesni@xula.edu; gzgolove@xula.edu

Xavier University of Louisiana, Chemistry Department, New Orleans, LA 70125

SUPPORTING INFORMATION

Content

Substance-in-use blank form for ionic compounds	S2
Substance-in-use blank form for elements	S3
Substance-in-use data sheet for dimethyl phthalate	S4
Substance-in-use data sheet for sodium hydride	S5
Substance-in-use data sheet for pyridinium tribromide	S6
Substance-in-use data sheet for 1,2-dichlorobenzene	S7
Substance-in-use data sheet for sulfanilic acid	S8
Substance-in-use data sheet for N,N'-dimethylaniline	S9
Substance-in-use data sheet for acetylacetone	S10
Selected quiz questions	S11
Selected test questions	S13

IONIC COMPOUND Chemical name and formula:

Formula weight:

Give the concise answers in the blank lines. Underline or highlight proper selections in the multiple-choice entries.

ROLE IN THIS EXPERIMENT:			
CHEMICAL PROPERTIES			
Solubility in water	Low	medium	high
Solubility in organic solvents (list)	Low:	Medium:	High:
	____________	____________	____________
	____________	____________	____________
Reactivity with water (give the details if reacts)			
Reactivity with oxygen			
Reactivity with moist CO2 at R.T.			
Reactivity with acids			
Reactivity with bases			
Reactivity with organic solvents			
Reactivity with neat oxidizing agents			
Thermal stability/decomposition			
HANDLING (check all that apply)	Hygroscopic<br>Air-sensitive Nitrogen blanket required<br>Fume hood required<br>Attacks rubber stoppers, tubing, bulbs, O-rings<br>Corrosive to metal-made lab equipment		
Spillage removing	Wiping Neutralizing		
Disposing	Waste bottle Neutralizing Trash Sink		
REACTION WORKUP (if this substance is to be removed)	Filter off Extract Neutralize (If so, how?)		
HAZARDS (Check all that apply)	Highly flammable Pyrophoric		
Violent reactivity	With water With acids With organic solvents		
TOXICITY	Low Moderate High		
Contact Hazard	Fumes inhalation Dust inhalation<br>Oral toxicity Skin irritation/burns		

ELEMENT Name and symbol:

Atomic weight:

Give the concise answers in the blank lines. Underline or highlight proper selections in the multiple-choice entries.

ROLE IN THIS EXPERIMENT	
PHYSICAL PROPERTIES<br>Melting point<br>Boiling point<br>Density<br>Volatility	 <br><br><br>low medium high
CHEMICAL PROPERTIES<br>Reactivity with water (give the details if reacts)<br>Reactivity with oxygen<br>Reactivity with halogens<br>Reactivity with acids<br>Reactivity with bases<br>Reactivity with metals<br>Reactivity with neat oxidizing agents<br>Reactivity with organic solvents<br>Solubility in organic solvents (list)	
HANDLING (check all that apply)	Air-sensitive Nitrogen blanket required<br>Fume hood required<br>Attacks rubber stoppers, tubing, bulbs, O-rings<br>Attacks rubber, latex or vinyl gloves.<br>Corrosive to metal-made lab equipment
Spillage removing	Wiping Neutralizing Allowing to evaporate
Disposing	Waste bottle Neutralizing Trash
REACTION WORKUP (if this substance is to be removed)	Evaporate Filter off Neutralize (if so, how?)
GLASSWARE CLEANING	Evaporate Wash away Neutralize
HAZARDS (Check all that apply)	Highly flammable Pyrophoric
Violent reactivity	With water With acids With metals<br>With organic solvents (list)
TOXICITY	Low Moderate High
Permissible exposure limit (PEL)*:	
Threshold limit value (TLV)*:	
Contact hazard	Vapor inhalation Dust inhalation Oral toxicity<br>Skin irritation/burns Irritant/lachrymator

*Refer to https://www.osha.gov/annotated-pels or other sources.

MOLECULAR COMPOUND   Chemical name and formula: *Dimethyl phthalate* $C_{10}H_{10}O_4$

Formula weight: *194.2*

Give the concise answers in the blank lines. Underline or highlight proper selections in the multiple-choice entries.

ROLE IN THIS EXPERIMENT: *Reagent*	
PHYSICAL PROPERTIES	
Melting point	*2*
Boiling point	*283*
Density	*1.19*
Polarity	low (highlighted) medium high
Volatility	low (highlighted) medium high
CHEMICAL PROPERTIES	
Solubility in water	low (highlighted) medium high
Solubility in organic solvents (list)	*Miscible with most organic solvents*
Reactivity with water (give the details if reacts)	*Very slow hydrolysis*
Reactivity with air at ambient conditions	*None*
Reactivity with organic solvents	*None*
Reactivity with acids	*Hydrolysis*
Reactivity with bases	*Hydrolysis*
Reactivity with alkali metals	*Reacts*
Reactivity with neat oxidizing agents	*Reacts*
Thermal stability/decomposition	*Stable*
HANDLING	□ Hygroscopic<br>□ Air-sensitive □ Nitrogen blanket required<br>□ Spillage removing:<br>Wiping (highlighted) Neutralizing Allowing to evaporate<br>□ Disposing:<br>Waste bottle (highlighted) Neutralizing Trash Sink<br>□ Fume hood required<br>□ Attacks rubber stoppers, tubing, bulbs, O-rings (highlighted)<br>□ Attacks rubber, latex or vinyl gloves. If so, select: Penetrates or destroys<br>□ Corrosive to metal-made lab equipment
REACTION WORKUP (if this substance is to be removed)	Evaporate Filter off Extract (highlighted)<br>Neutralize (if so, how?)
GLASSWARE CLEANING	Evaporate Wash away (highlighted) Neutralize
HAZARDS (Check all that apply)<br>______________________<br>Permissible exposure limit (PEL):<br><br>Threshold limit value (TLV):<br><br>https://www.osha.gov/annotated-pels	□ Highly flammable □ Pyrophoric<br>□ <u>Violent reactivity</u>: With water With acids<br>With bases With organic solvents (list):<br><br>□ <u>Contact hazard</u>: skin irritation/burns<br>□ <u>Toxicity</u>: low (highlighted) moderate high<br>Vapor inhalation Dust inhalation<br>Skin penetration (highlighted) Oral toxicity<br>□ Irritant/lachrymator

IONIC COMPOUND Chemical name and formula: *Sodium hydride NaH*

Formula weight: 24.0

Give the concise answers in the blank lines. Underline or highlight proper selections in the multiple-choice entries.

ROLE IN THIS EXPERIMENT: *Reagent*	
CHEMICAL PROPERTIES	
Solubility in water	Low medium high
Solubility in organic solvents (list)	Low: Medium: High:<br>____ ____ ____<br>____ ____ ____
Reactivity with water (give the details if reacts)	*Violent hydrolysis*
Reactivity with oxygen	*Yes*
Reactivity with moist CO2 at R.T.	*Yes*
Reactivity with acids	*Yes*
Reactivity with bases	*Yes – with aqueous bases*
Reactivity with organic solvents	*Yes – protic solvents*
Reactivity with neat oxidizing agents	*Reacts*
Thermal stability/decomposition	*Stable*
HANDLING	□ Hygroscopic<br>□ Air-sensitive □ Nitrogen blanket required<br><br>□ Spillage removing:<br>Wiping Neutralizing<br><br>□ Disposing:<br>Waste bottle Neutralizing Trash Sink<br><br>□ Fume hood required<br><br>□ Attacks rubber stoppers, tubing, bulbs, O-rings<br><br>□ Corrosive to metal-made lab equipment
REACTION WORKUP (if this substance is to be removed)	Filter off Extract Neutralize (If so, how?) Methanol, ethanol, etc.
HAZARDS Check all that apply:	□ Highly flammable □ Pyrophoric<br>□ Violent reactivity: With water<br>□ Violent reactivity: With acids<br>□ Violent reactivity: With organic solvents<br>□ Contact hazard: skin irritation/burns<br>□ Toxicity: low moderate high<br>Fumes inhalation<br>Dust inhalation<br>Oral toxicity

IONIC COMPOUND   Chemical name and formula: *Pyridinium tribromide $C_5H_6Br_3N$*

Formula weight: 319.9

Give the concise answers in the blank lines. Underline or highlight proper selections in the multiple-choice entries.

ROLE IN THIS EXPERIMENT: *Reagent*	
CHEMICAL PROPERTIES	
Solubility in water	Low medium high
Solubility in organic solvents (list)	Low: CH_2Cl_2, EtOAc<br>Medium: ______ ______<br>High: HOAc, EtOH, CH_3CN, glyme
Reactivity with water (give the details if reacts)	*Very slow*
Reactivity with oxygen	*None*
Reactivity with moist CO2 at R.T.	*Very slow*
Reactivity with acids	*None*
Reactivity with bases	*Yes*
Reactivity with organic solvents	*Yes – bromination*
Reactivity with neat oxidizing agents	*Reacts*
Thermal stability/decomposition	*Unstable – store in refrigerator*
HANDLING	□ Hygroscopic<br>□ Air-sensitive □ Nitrogen blanket required<br>□ Spillage removing: Wiping Neutralizing<br>□ Disposing: Waste bottle Neutralizing Trash Sink<br>□ Fume hood required<br>□ Attacks rubber stoppers, tubing, bulbs, O-rings<br>□ Corrosive to metal-made lab equipment
REACTION WORKUP (if this substance is to be removed)	Filter off Extract Neutralize (If so, how?) Aqueous $Na_2S_2O_3$
HAZARDS Check all that apply:	□ Highly flammable □ Pyrophoric<br>□ Violent reactivity: With water<br>□ Violent reactivity: With acids<br>□ Violent reactivity: With organic solvents<br>□ Contact hazard: skin irritation/burns<br>□ Toxicity: low moderate high<br>Fumes inhalation<br>Dust inhalation<br>Oral toxicity

MOLECULAR COMPOUND Chemical name and formula: *1,2-dichlorobenzene* $C_6H_4Cl_2$

Formula weight: *147.0*

Give the concise answers in the blank lines. Underline or highlight proper selections in the multiple-choice entries.

ROLE IN THIS EXPERIMENT: *Solvent*	
PHYSICAL PROPERTIES	
Melting point	*-17*
Boiling point	*180.2*
Density	*1.30*
Polarity	low medium high
Volatility	low medium high
CHEMICAL PROPERTIES	
Solubility in water	low medium high
Solubility in organic solvents (list)	*Miscible with most organic solvents*
Reactivity with water (give the details if reacts)	*None*
Reactivity with air at ambient conditions	*None*
Reactivity with organic solvents	*None*
Reactivity with acids	*None*
Reactivity with bases	*None*
Reactivity with alkali metals	*Reacts*
Reactivity with neat oxidizing agents	*Thermodynamically – yes, but practically – no (kinetics)*
Thermal stability/decomposition	*Stable*
HANDLING	□ Hygroscopic<br>□ Air-sensitive □ Nitrogen blanket required<br>□ Spillage removing:<br>Wiping Neutralizing Allowing to evaporate<br>□ Disposing:<br>Waste bottle Neutralizing Trash Sink<br>□ Fume hood required<br>□ Attacks rubber stoppers, tubing, bulbs, O-rings<br>□ Attacks rubber, latex or vinyl gloves. If so, select:<br>Penetrates or destroys<br>□ Corrosive to metal-made lab equipment
REACTION WORKUP (if this substance is to be removed)	Evaporate Filter off Extract<br>Neutralize (if so, how?)
GLASSWARE CLEANING	Evaporate Wash away Neutralize
HAZARDS (Check all that apply)<br><br>Permissible exposure limit (PEL): *50 ppm*<br><br>Threshold limit value (TLV):<br><br>https://www.osha.gov/annotated-pels	□ Highly flammable □ Pyrophoric<br>□ Violent reactivity: With water With acids<br>With bases With organic solvents (list):<br><br>□ Contact hazard: skin irritation/burns<br>□ Toxicity: low moderate high<br>Vapor inhalation Dust inhalation<br>Skin penetration Oral toxicity<br>□ Irritant/lachrymator

IONIC COMPOUND   Chemical name and formula: *Sulfanilic acid $C_6H_7NO_3S$*

Formula weight: 173.2

Give the concise answers in the blank lines. Underline or highlight proper selections in the multiple-choice entries.

ROLE IN THIS EXPERIMENT: *Reagent*	
CHEMICAL PROPERTIES	
Solubility in water	Low ==medium== high
Solubility in organic solvents (list)	==Low==: *Methanol* *Ethanol* Medium: ____ ____ High: ____ ____
Reactivity with water (give the details if reacts)	*pKa 3.23*
Reactivity with oxygen	*None*
Reactivity with moist CO2 at R.T.	*None*
Reactivity with acids	*Protonated*
Reactivity with bases	*Deprotonated*
Reactivity with organic solvents	*None*
Reactivity with neat oxidizing agents	*Reacts*
Thermal stability/decomposition	*Stable*
HANDLING	□ Hygroscopic<br>□ Air-sensitive □ Nitrogen blanket required<br>□ Spillage removing:<br>==Wiping== Neutralizing<br>□ Disposing:<br>==Waste bottle== Neutralizing Trash Sink<br>□ Fume hood required<br>□ Attacks rubber stoppers, tubing, bulbs, O-rings<br>□ Corrosive to metal-made lab equipment
REACTION WORKUP (if this substance is to be removed)	Filter off Extract ==Neutralize== (If so, how?)<br>Water, aq. Acid or base
HAZARDS Check all that apply:	□ Highly flammable □ Pyrophoric<br>□ Violent reactivity: With water<br>□ Violent reactivity: With acids<br>□ Violent reactivity: With organic solvents<br>□ <u>Contact hazard</u>: skin irritation/burns<br>□ <u>Toxicity</u>: low moderate high<br>Fumes inhalation<br>Dust inhalation<br>==Oral toxicity==

MOLECULAR COMPOUND   Chemical name and formula: *N,N'-dimethylaniline* $C_8H_{11}N$
Formula weight: *121.2*

Give the concise answers in the blank lines. Underline or highlight proper selections in the multiple-choice entries.

ROLE IN THIS EXPERIMENT: *Reagent*	
PHYSICAL PROPERTIES	
Melting point	*2*
Boiling point	*194*
Density	*0.956*
Polarity	low medium high
Volatility	low medium high
CHEMICAL PROPERTIES	
Solubility in water	low medium high
Solubility in organic solvents (list)	*Miscible with most organic solvents*
Reactivity with water (give the details if reacts)	*pH↑*
Reactivity with air at ambient conditions	*None*
Reactivity with organic solvents	*None*
Reactivity with acids	*Protonation*
Reactivity with bases	*None*
Reactivity with alkali metals	*none*
Reactivity with neat oxidizing agents	*Reacts*
Thermal stability/decomposition	*Stable*
HANDLING	□ Hygroscopic<br>□ Air-sensitive □ Nitrogen blanket required<br>□ Spillage removing:<br>Wiping Neutralizing Allowing to evaporate<br>□ Disposing:<br>Waste bottle Neutralizing Trash Sink<br>□ Fume hood required<br>□ Attacks rubber stoppers, tubing, bulbs, O-rings<br>□ Attacks rubber, latex or vinyl gloves. If so, select:<br>Penetrates or destroys<br>□ Corrosive to metal-made lab equipment
REACTION WORKUP (if this substance is to be removed)	Evaporate Filter off Extract<br>Neutralize (if so, how?) *aqueous acid*
GLASSWARE CLEANING	Evaporate Wash away Neutralize
HAZARDS (Check all that apply)<br><br>Permissible exposure limit (PEL): *5 ppm*<br><br>Threshold limit value (TLV):<br><br>https://www.osha.gov/annotated-pels	□ Highly flammable □ Pyrophoric<br>□ Violent reactivity: With water With acids<br>With bases With organic solvents (list):<br><br>□ Contact hazard: skin irritation/burns<br>□ Toxicity: low moderate high<br>Vapor inhalation Dust inhalation<br>Skin penetration Oral toxicity<br>□ Irritant/lachrymator

MOLECULAR COMPOUND   Chemical name and formula: *acetylacetone* $C_5H_8O_2$

Formula weight: *100.1*

Give the concise answers in the blank lines. Underline or highlight proper selections in the multiple-choice entries.

ROLE IN THIS EXPERIMENT: *Reagent*	
PHYSICAL PROPERTIES	
Melting point	*-23*
Boiling point	*140*
Density	*0.975*
Polarity	low medium ==high==
Volatility	low ==medium== high
CHEMICAL PROPERTIES	
Solubility in water	low medium ==high==
Solubility in organic solvents (list)	*Miscible with most organic solvents*
Reactivity with water (give the details if reacts)	*Keto/enol shift*
Reactivity with air at ambient conditions	*None*
Reactivity with organic solvents	*None*
Reactivity with acids	*None*
Reactivity with bases	*Deprotonation*
Reactivity with alkali metals	*Reacts*
Reactivity with neat oxidizing agents	*Reacts*
Thermal stability/decomposition	*Stable*
HANDLING	□ Hygroscopic<br>□ Air-sensitive □ Nitrogen blanket required<br>□ Spillage removing:<br>==Wiping== Neutralizing Allowing to evaporate<br>□ Disposing:<br>==Waste bottle== Neutralizing Trash Sink<br>□ ==Fume hood required==<br>□ ==Attacks rubber stoppers, tubing, bulbs, O-rings==<br>□ ==Attacks rubber, latex or vinyl gloves==. If so, select: ==Penetrates== or destroys<br>□ Corrosive to metal-made lab equipment
REACTION WORKUP (if this substance is to be removed)	==Evaporate== Filter off ==Extract==<br>Neutralize (if so, how?)
GLASSWARE CLEANING	==Evaporate== ==Wash away== Neutralize
HAZARDS (Check all that apply)<br><br>Permissible exposure limit (PEL):<br><br>Threshold limit value (TLV): *25 ppm*<br><br>https://www.osha.gov/annotated-pels	□ ==Highly flammable== □ Pyrophoric<br>□ Violent reactivity: With water With acids<br>With bases With organic solvents (list):<br><br>□ Contact hazard: skin irritation/burns<br>□ Toxicity: low ==moderate== high<br>==Vapor inhalation== Dust inhalation<br>==Skin penetration== Oral toxicity<br>□ Irritant/lachrymator

SELECTED QUIZ QUESTIONS:

What is the role of toluene in this experiment? What properties make it useful in this experiment?

How do the physical properties of toluene relate to its structure?

How are the chemical properties of toluene related to its structure?

Underline the toxic exposure path(s) of toluene.

Vapor inhalation	Dust inhalation
Skin penetration	Oral toxicity

List the properties of toluene which determine its toxic exposure path(s).

Choose hazards associated with bromine. Highly flammable. Pyrophoric. Highly toxic. Corrosive. Skin burns. Vapor inhalation. Dust inhalation. Oral toxicity. Irritant. Violently reacts with water.

In the synthesis workup procedure, what is (are) the way to remove aluminum chloride catalyst (explain)?

- Evaporate
- Hydrolyze
- Oxidize
- Reduce
- Distill off
- Filter off
- Wash away

In the synthesis workup procedure, what is (are) the way to remove toluene?

- Evaporate
- Hydrolyze
- Oxidize
- Reduce
- Distill off
- Filter off
- Wash away

In the synthesis workup procedure, what is (are) the way to remove bromine?

- Evaporate
- Hydrolyze
- Oxidize
- Reduce
- Distill off
- Filter off
- Wash away

The best sequence of steps to contain toluene spilled in the fume hood, is (use the numbering 1, 2, 3, …).

- Use paper towels to absorb the liquid and transfer in the empty jar
- Remove hot plate(s) from the hood
- Extinguish any open flame
- Leave it untouched and allow to evaporate
- Put vinyl gloves on

The right method(s) for bromine disposal is (are):

- Pour it in “halogenated waste” bottle
- Pour it in “non-halogenated waste” bottle
- Neutralize it with water and pour down the drain
- Mix it with sodium thiosulfate solution and pour down the drain

SELECTED TEST QUESTIONS:

There are three types of data sheets provided for condensed-phase chemicals based on their composition and structure: the elements (E), molecular (M) and ionic compounds (I). Associate each listed below with one or another, E, M or I.

- Sulfuric acid
- Sodium hydroxide
- Chlorobenzene
- Aluminum chloride
- Pyridine
- Iodine

Connect each of the following physical properties to the type of the Data Sheets where they are listed by indicating E, M or I. For the properties listed in more than one category indicate all. Briefly justify.

- Volatility
- Polarity
- Density
- Melting point
- Boiling point

What is (are) the common toxic exposure path(s) for molecular substances? (select).

Vapor inhalation	Dust inhalation
Skin penetration	Oral toxicity

What is (are) the common toxic exposure path(s) for ionic compounds? (select).

Vapor inhalation	Dust inhalation
Skin penetration	Oral toxicity

Fill in blanks: A cylinder which was used to measure and deliver ________ does not have to be washed. To what substances, other than water, is this applicable (select and list)? Sulfuric acid, acetic acid, toluene, methanol, chloroform, MTBE, ethyl acetate, dimethyl sulfoxide.

Which of the following chemicals is (are) highly flammable? Sulfuric acid, sodium hydroxide, sodium, aluminum chloride, bromine, sodium hydride.

Which of the following chemicals violently react with water? Sulfuric acid, sodium hydroxide, sodium, aluminum chloride, bromine, sodium hydride.

Which of the following chemicals react with oxygen at room temperature? Sulfuric acid, sodium hydroxide, sodium, aluminum chloride, bromine, sodium hydride.

Which of the following chemicals is (are) highly corrosive? Sulfuric acid, sodium hydroxide, sodium, aluminum chloride, bromine, sodium hydride.

Dimethylmercury and diphenylmercury share the same structure of linear molecules. Their normal boiling points are 93 and 204°C, respectively. Which one is more likely to be toxicity hazardous?

Lead organic compounds $PbMe_4$ and $PbEt_4$ (was used as a gasoline additive) share the same structure of tetrahedral molecules. They are liquids with normal boiling points 110 and 195 °C, respectively. Which one is more likely to be toxicity hazardous?

Phenyllead $PbPh_4$ is a nonvolatile powdery solid with tetrahedral molecular structure like lead alkyls. Is it okay to work with it out of fume hood on the bench? Explain.

Polarity is included among physical properties in the Data Sheet for molecular but not for ionic compounds or elements. Explain.

Volatility is included among physical properties in the Data Sheet for elements and molecular but not for ionic compounds. Explain.
